# Efficacy comparison of moderate to severe astigmatism: implantable collamer lens combined with paired opposite clear corneal incisions versus toric implantable collamer lens

**DOI:** 10.1186/s12886-026-04948-w

**Published:** 2026-05-29

**Authors:** Xiaoguang Zhang, Yangyan Wan, Weixing Wang

**Affiliations:** 1 Nanchang Honggutan Bright Eye Hospital, Nanchang, Jiangxi China; 2 Nanchang Hongcheng Aier Eye Hospital, Commercial Building No. 3, Laimengduhui Commercial Center (Plot B-14), Honggutan District, Nanchang City, Jiangxi Province China

**Keywords:** Implantable collamer lens, Toric implantable collamer lens, Moderate to severe astigmatism, Paired opposite clear corneal incision

## Abstract

**Purpose:**

To compare the efficacy of Implantable Collamer Lens (ICL) combined with paired opposite clear corneal incisions (OCCI) versus Toric Implantable Collamer Lens (TICL) for moderate to severe astigmatism.

**Methods:**

This retrospective comparative study included 40 patients with moderate to severe astigmatism (cylinder 1·5D-3·0D). Based on patient preference, 20 eyes underwent TICL implantation (TICL group) and 20 eyes underwent ICL with OCCI (OCCI group). Preoperative and 6-month postoperative data were analyzed, including uncorrected and corrected distance visual acuity (UDVA, CDVA), refraction, higher-order aberrations (HOAs), and Alpins vector parameters (target-induced astigmatism [TIA], surgically induced astigmatism [SIA], difference vector [DV], correction index [CI], angle of error [AE]).

**Results:**

At 6 months postoperatively, both groups showed significant visual improvement. The proportion achieving UDVA ≥ 20/20 was 100% in TICL group and 95% in OCCI group. Residual astigmatism ≤ 1·0D was achieved in 95% (TICL) and 90% (OCCI). Spherical equivalent within ±1·0D was 95% and 90%, respectively. OCCI induced significantly more HOAs, horizontal coma, and horizontal trefoil postoperatively compared to TICL (all *p* < 0.05). Vector analysis showed no significant between-group differences in TIA, SIA, DV, CI, or AE (all *p* > 0.05).

**Conclusions:**

ICL combined with OCCI yields acceptable visual and refractive outcomes for moderate to severe astigmatism, though slightly inferior to TICL in accuracy and HOAs. It represents a viable alternative when TICL is unavailable or contraindicated.

## Introduction

Astigmatism is one of the main factors affecting visual quality. More than 0.75 diopters (D) of astigmatism can cause blurred vision, decreased vision, and visual fatigue. Currently, the main treatment methods for astigmatism are wearing glasses, refractive surgery including keratorefractive surgery, toric intraocular lens (IOL) implantation, and corneal relaxing incisions (CRI).

Toric Implantable Collamer Lens (TICL; STAAR Surgical) has been widely used in the treatment of high myopic astigmatism, and several studies have confirmed that TICL could achieve good results in the correction of moderate and high myopic astigmatism [1–3]. However, it still has its own shortcomings, such as long waiting time for the lens, higher cost compared to Implantable Collamer Lens (ICL), and the risk of rotation which may cause reoperation to adjust the axial position, leading to poor satisfaction of patients.

CRI can correct corneal astigmatism by changing the location, size, shape, depth, and number of the incisions [4, 5]. At present, it has been widely used to correct astigmatism in cataract surgery [6–9]. Unilateral clear corneal incision (CCI) and opposite clear corneal incision (OCCI) are common approaches classified under CRI. Previously, there were reports of CCI combined with ICL and astigmatism keratotomy (AK) combined with ICL for the treatment of myopic astigmatism, which proved to be an effective method to correct astigmatism [10–12]. Zhou et al. have reported that OCCI combined with ICL had a certain effect on mild to moderate astigmatism [13]. Previous studies on OCCI combined with ICL have primarily focused on low to moderate astigmatism, often using lamellar rather than full-thickness incisions. The application of bilateral penetrating OCCI with ICL for moderate to severe astigmatism (1.5–3.0 D) remains rarely reported, and no prior study has directly compared this approach with TICL implantation. Given that some patients may decline TICL because of cost, waiting time, or rotation risk, it is clinically relevant to assess whether ICL with paired penetrating OCCI can provide acceptable efficacy relative to the TICL benchmark. Accordingly, this study compared the safety and efficacy of these two approaches for correcting moderate to severe astigmatism.

## Patients and methods

This was a retrospective comparative study. The medical records of 40 patients with moderate to severe astigmatism who underwent refractive surgery at Nanchang Honggutan Bright Eye Hospital between September 2022 and December 2024 were retrospectively reviewed. Patients were divided into two groups according to their preference after thorough discussion of the risks and benefits of each procedure: 20 patients who chose Toric Implantable Collamer Lens implantation (TICL group) and 20 patients who chose ICL implantation combined with paired opposite clear corneal incisions (OCCI group). Only one eye per patient was included in the study. The study was approved by the Ethics Committee of our hospital and conducted in accordance with the Declaration of Helsinki. Due to the retrospective nature of the study, the requirement for informed consent was waived. Inclusion criteria were: age over 18 years, normal ocular examination findings, stable refraction for more than 2 years, cylindrical power between 1.5 D and 3.0 D, anterior chamber depth > 2.8 mm and corneal endothelial cell density > 2300 cells/mm2. Patients with ocular inflammatory diseases, previous ocular surgery or trauma, corneal diseases such as pterygium, and other diseases unsuitable for surgery were excluded.

### Preoperative and postoperative examination

Before surgery, and at 1 week, 1, 3, and 6 months postoperatively, all patients underwent the following ophthalmic examinations, including uncorrected distance visual acuity (UDVA), corrected distance visual acuity (CDVA), subjective refraction, slit lamp examination, corneal topography (Pentacam, Oculus, Germany), and intraocular pressure. UDVA and CDVA were examined by Snellen charts and were converted to the logarithm of the minimum angle of resolution (logMAR) for analysis. Corneal endothelial cell count, ultrasound biomicroscopy (UBM, Suoer, China), B-scan ocular ultrasonography, fundus photography and macular OCT were also required to rule out any contraindications to surgery. Anterior chamber depth, corneal diameter, corneal curvature, total higher order aberrations (HOAs), spherical aberration $Z_{4}^{0}$, horizontal coma $Z_{3}^{1}$, vertical coma $Z_{3}^{-1}$, horizontal trefoil $Z_{3}^{3}$ and oblique trefoil $Z_{3}^{-3}$ were obtained by Pentacam. Higher order aberrations were recorded within a 6 mm central corneal zone. For the analysis of higherorder aberrations, only eyes with Pentacam images meeting the instrument’s quality standards (adequate centration, regular tear film, and complete data capture within the 6 mm zone) at the 6 month followup were included; eyes with poor image quality were excluded to ensure the reliability of the aberration measurements. vertic

### Analysis of astigmatism correction

Alpins astigmatism vector analysis was used to analyze target-induced astigmatism (TIA), surgically induced astigmatism (SIA), difference vector (DV), correction index (CI), and angle of error (AE). The refractive analysis function was selected in the online Alpins software (https://lasikbbs.com/). TIA is the target-induced astigmatism magnitude. SIA is the surgically induced astigmatism magnitude. DV is the difference vector. When TIA is zero, DV is equal to the postoperative astigmatism. CI is the ratio of SIA to TIA. CI = 1 indicates that the targeted astigmatism is completely corrected; CI > 1 indicates overcorrection; CI < 1 indicates undercorrection.

### Surgical procedure

Patients were seated in front of a slit lamp before surgery, and 1 mm syringe needles were used for corneal marking. Both groups were operated under topical anesthesia by using 0.5% proparacaine hydrochloride eye drops (Alcaine, Alcon, USA). In the TICL group, the preoperative corneal astigmatism axes were marked according to the rotational chart recommended by STAAR Surgical. In the OCCI group, the steep corneal axes were marked bilaterally according to the subjective refraction. In the TICL group, the surgical procedures were routine. In short, a 2.8 mm incision was made 1 mm anterior to the upper limbus. The TICL was implanted and its astigmatic axis was aligned with the preoperative corneal markers. Finally, the incision was hydrated to ensure a watertight seal. In the OCCI group, a 3 mm major incision was made 1 mm anterior to the preoperatively marked corneal limbus. The ICL was placed behind the iris and rotated to the correct position. Before rinsing off the sodium hyaluronate, a 3 mm penetrating incision was made 1 mm anterior to the contralateral limbal at the opposite marked meridian. According to the magnitude of astigmatism, the bilateral incisions were expanded appropriately as follows: 3.0 mm/3.0 mm for −1.5D; 3.0 mm/3.5 mm for −2.0D; 3.5 mm/3.5 mm for −2.5D to −3.0D. Finally, the incisions were hydrated to achieve a watertight closure.

### Statistical analysis

SPSS software (version 27.0.0; IBM Corporation) was used for statistical analysis. Measurement data were expressed as mean ± standard deviation. According to the data type, the corresponding statistical method was selected. Frequency histograms were used to test whether the data followed a normal distribution. The matched samples t-test was used to compare differences in means for the same samples under different conditions. The independent samples t-test was used to compare the difference in means between the two independent samples. *p* < 0.05 was considered statistically significant.

## Results

### Basic characteristics

As shown in Table 1, there were 20 patients each in the TICL and OCCI groups. One eye per patient was included in the analysis. The proportion of men and women in each group was coincidentally the same, 45 and 55%, respectively. Both the TICL and OCCI groups had the same age range, 18 to 41 years. There were no significant differences in spherical diopter, cylindrical power, SEQ, UDVA (LogMAR) or CDVA (LogMAR) between the two groups before surgery.

**Table 1:** Comparison of preoperative and postoperative demographic data between the TICL and OCCI groups

Characteristic	TICL Group	OCCI Group	*P* ^*b*^
Patients (eyes, n)	20, 20	20, 20	-
Age (y)^a^	25.3 ± 3.4 (18 to 41)	26.9 ± 7.2 (18 to 41)	0.38
Sex (female/male)	45％/55％	45％/55％	-
Preoperative			
Spherical(D)^a^	−7.99 ± 3.47 (−3.50 to −16.00)	−8.01 ± 2.77 (−3.25 to −13.50)	0.98
Cylindrical(D)^a^	−1.96 ± 0.27 (−1.75 to −2.50)	−1.91 ± 0.26 (−1.50 to −2.50)	0.56
SEQ (D)^a^	−8.97 ± 3.52 (−4.38 to −17.25)	−8.97 ± 2.78 (−4.25 to −14.38)	1.00
UDVA (logMAR)^a^	1.49 ± 0.28 (1.0 to 1.70)	1.33 ± 0.27 (0.8 to 1.70)	0.08
CDVA (logMAR)^a^	0.05 ± 0.05 (0 to 0.15)	0.03 ± 0.05 (0 to 0.10)	0.28
Postoperative 6 months			
Spherical(D)^a^	0.29 ± 0.47 (−1.00 to 1.00)*	0.11 ± 0.43 (−1.00 to 0.75)*	0.23
Cylindrical(D)^a^	−0.60 ± 0.26 (−1.25 to −0.25)*	−0.66 ± 0.36 (−1.25 to 0)*	0.53
SEQ (D)^a^	−0.01 ± 0.52 (−1.63 to 0.625)*	−0.22 ± 0.46 (−1.38 to 0.5)*	0.19
UDVA (logMAR)^a^	−0.06 ± 0.05 (−0.18 to 0)*	−0.04 ± 0.05 (−0.08 to 0)*	0.38

D = diopters; UDVA = uncorrected distance visual acuity; CDVA = corrected distance visual acuity; SEQ = spherical equivalent

refraction; OCCI = opposite clear corneal incisions

^a^ Values are presented as mean ± standard deviation (range)

* Indicates significant difference between preoperative and postoperative values (＜0.05)

^b^ Means comparison between the TICL and OCCI groups

### Visual acuity and refractive outcomes

**Fig. 1:**
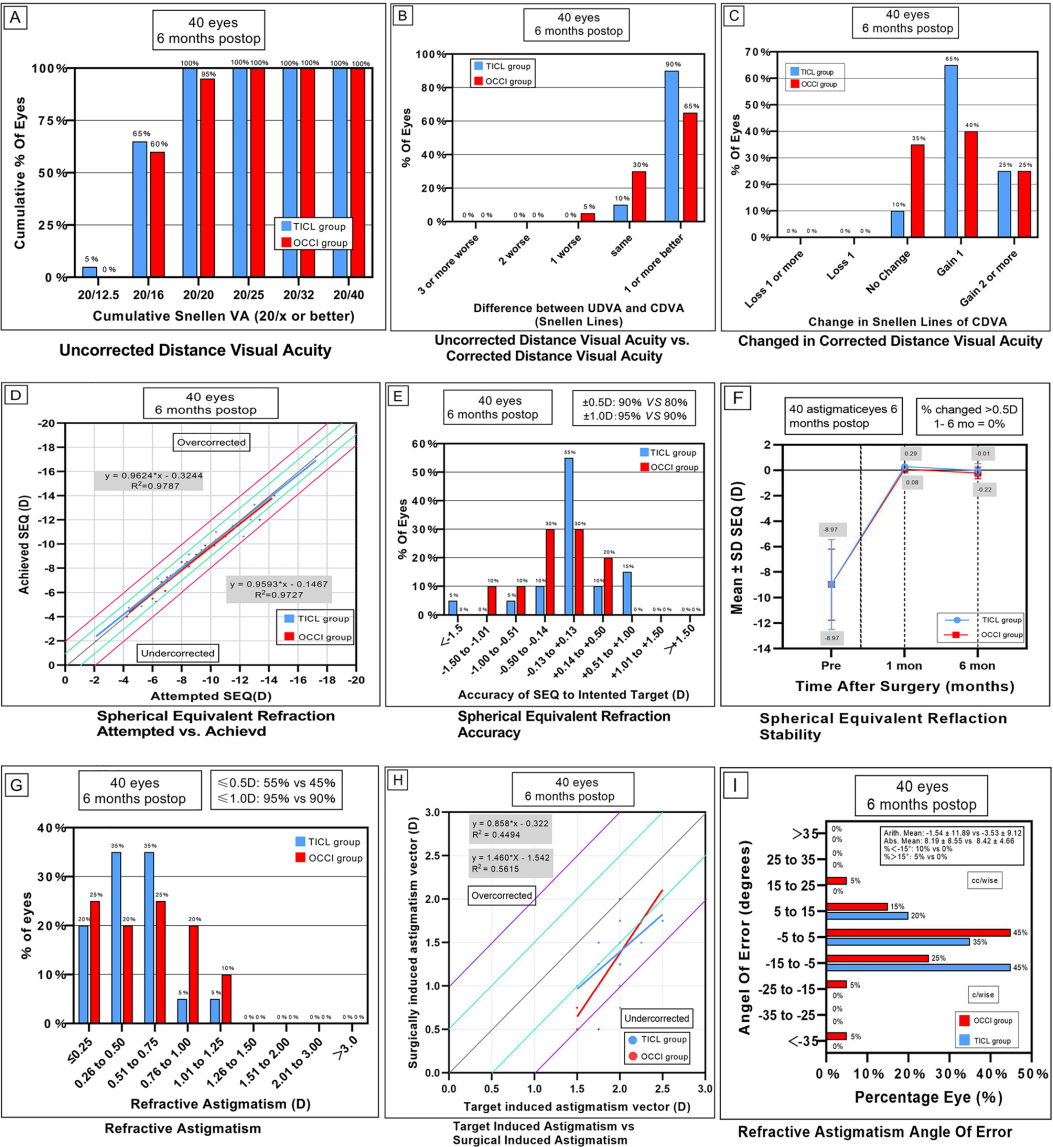
Visual and refractive outcomes after TICL implantation and ICL combined with OCCI. (**A**) Postoperative uncorrected distance visual acuity (UDVA) distribution (≥20/20). (**B**) Proportion of eyes with postoperative UDVA improved by one line or more compared with preoperative corrected distance visual acuity (CDVA). (**C**) Change in CDVA from preoperative to 6 months postoperatively. (**D**) Linear regression analysis of attempted versus achieved spherical equivalent (SEQ). (**E**) Refractive accuracy: percentage of eyes with SEQ within ±0.50 D and ±1.00 D. (**F**) Stability of SEQ at 1 and 6 months postoperatively. (**G**) Residual refractive astigmatism within 0.50 D and 1.00 D. (**H**) Linear regression of target‑induced astigmatism (TIA) versus surgically induced astigmatism (SIA). (**I**) Distribution of angle of error (AE) at 6 months. D = diopters; logMAR = logarithm of the minimum angle of resolution

At 6 months after operation, the spherical, cylindrical and SEQ values of the two groups were significantly decreased compared to those before operation, and the UDVA was significantly better than that before operation. However, there were no significant differences in spherical diopter, cylindrical power, SEQ and UDVA between the two groups at 6 months after surgery. These data are summarized in Table 1. The proportions of patients achieving visual acuity of 20/20 or better in the TICL group and the OCCI group were 100 and 95%, respectively (Fig. 1A). The proportions of patients in the TICL and the OCCI groups whose postoperative UDVA improved by one line compared to their preoperative CDVA were 90 and 65%, respectively (Fig. 1B). In both groups, the proportion of patients whose postoperative CDVA lost one line or more compared with preoperative CDVA was 0, while the proportion of those in the TICL group and the OCCI group whose postoperative CDVA improved by one line were 65 and 40%, respectively (Fig. 1C). The linear regression analysis of SEQ demonstrated good predictability in the OCCI group, comparable to the TICL group (Fig. 1D). The R^2^ values of both the OCCI group and the TICL group were close to 1, indicating that the SEQ predictability of the OCCI group was accurate and there was almost no difference compared with the TICL group (Fig. 1D). The proportions of postoperative SEQ within ±0.5D in the TICL group and the OCCI group were 90 and 80% respectively, while the proportions within ±1.0D were 95 and 90% respectively (Fig. 1E). The SEQ remained basically stable one month and six months after the surgery (Fig. 1F). The proportions of postoperative residual astigmatism within 0.5D in the TICL group and the OCCI group were 55 and 45% respectively, and the proportions within 1.0D were 95 and 90% respectively (Fig. 1G). The linear regression analysis of TIA and SIA showed that the R^2^ values of both were relatively close (Fig. 1H). The arithmetic mean values of the astigmatism angle error in the OCCI group and the TICL group were −1.54 ± 11.89 and −3.53 ± 9.12 respectively, the absolute mean values were 8.19 ± 8.55 and 8.42 ± 4.66 respectively, the proportions less than −15° were 10 and 0% respectively, and the proportions greater than 15° were 5 and 0% respectively (Fig. 1I).

### Vector analysis

**Fig. 2:**
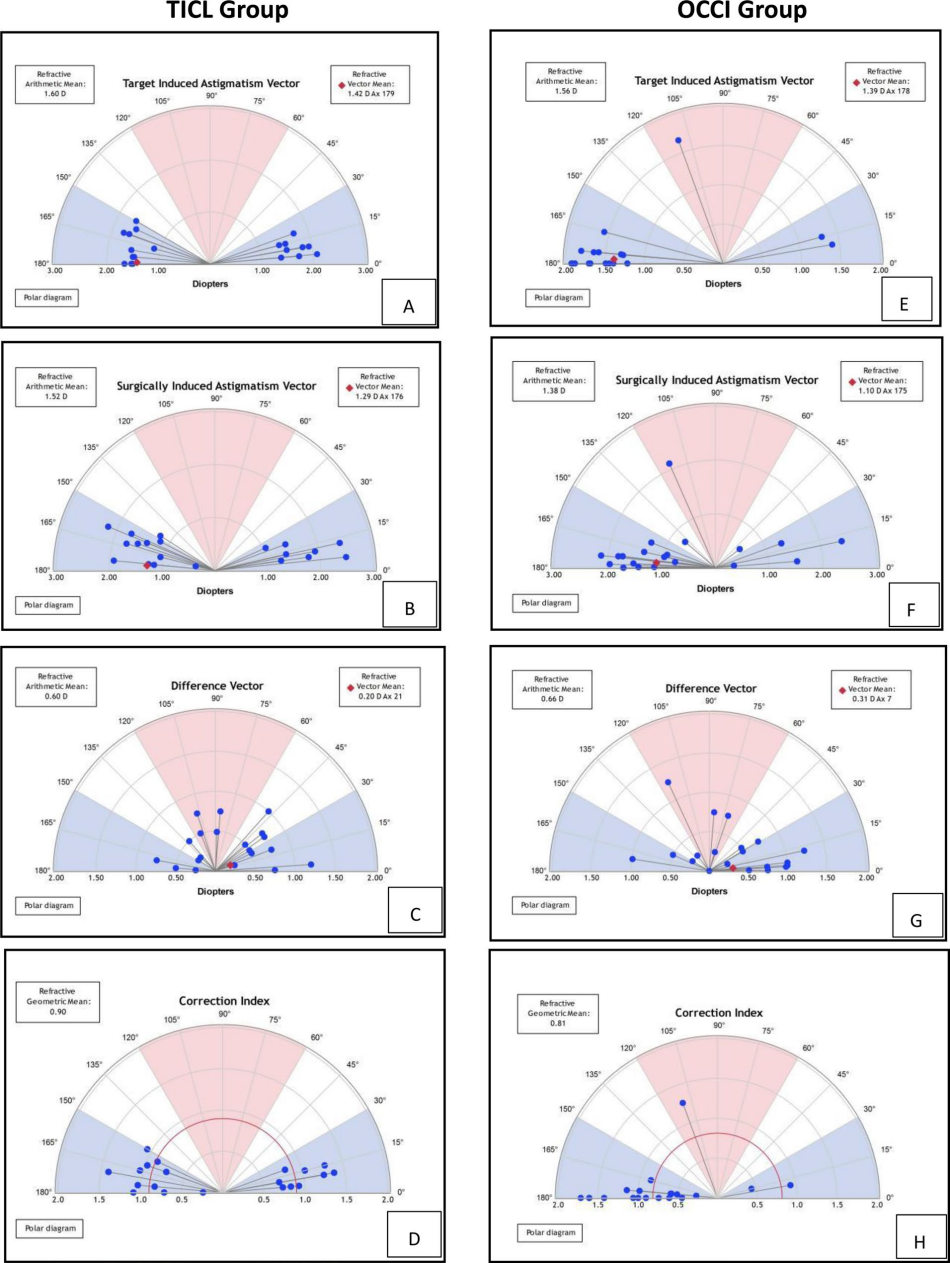
Single‑angle polar plots of vector parameters for the TICL and OCCI groups at 6 months postoperatively. (**A**–**D**) TICL group: (**A**) target‑induced astigmatism (TIA), (**B**) surgically induced astigmatism (SIA), (**C**) difference vector (DV), (**D**) correction index (CI). (**E**–**H**) OCCI group: (**E**) TIA, (**F**) SIA, (**G**) DV, (**H**) CI. Vector means are plotted as red diamonds

The results of Alpins vector analysis at 6 months post-surgery are presented in Table 2 and Fig. 2. As shown in Table 2, the mean values of TIA in the TICL group and OCCI group were 1.60 ± 0.21 and 1.56 ± 0.23, respectively, while the mean values of SIA in the TICL group and OCCI group were 1.52 ± 0.51 and 1.38 ± 0.56, respectively. Additionally, the mean values of DV in TICL group and OCCI group were 0.60 ± 0.26 and 0.66 ± 0.35, respectively, and the mean values of CI for the same groups were 0.95 ± 0.27 and 0.90 ± 0.40, respectively. There were no significant differences in TIA, SIA, DV, CI and AE between the TICL and OCCI groups. Figure 2 shows the single-angle plots of SIA, TIA, DV and CI, including their respective arithmetic means, vector means, and detailed scatter plots.

**Table 2:** Astigmatism vector analysis results after TICL and OCCI at 6 months^a^

Parameter	TICL (20 eyes)	OCCI (20 eyes)	*t*	*P*
TIA (D)	1.60 ± 0.21	1.56 ± 0.23	0.563	0.577
Vector	1.42&179	1.39&178		
Postoperative (6 months)				
SIA (D)	1.52 ± 0.51	1.38 ± 0.56	0.815	0.42
Vector	1.29&176	1.10&175		
DV (D)	0.60 ± 0.26	0.66 ± 0.35	−0.610	0.546
Vector	0.20&21	0.31&7		
CI	0.95 ± 0.27	0.90 ± 0.40	0.449	0.656
│AE│	8.42 ± 4.66	8.19 ± 8.55	0.107	0.916

TICL = Toric Implantable Collamer Lens (STAAR Surgical);OCCI = opposite clear corneal incision; TIA = target induced astigmatism; SIA = surgically induced astigmatism; DV = difference vector; AE = angle of error; CI = correction index; D = diopters; ME = magnitude of error^a^ Values presented as means ± standard deviation; &=diopters Ax

### Corneal aberration variations

Table 3 shows the corneal aberration variations between preoperative and postoperative 6 months. As can be seen from Table 3, there was no significant difference in preoperative HOAs, spherical aberration, coma or trefoil. At postoperative 6 months, the OCCI group had significantly more HOAs and $Z_{3}^{3}$ than the TICL group. The HOAs, $Z^{1}_{3}$, and $Z_{3}^{3}$ were significantly increased compared to before surgery in the OCCI group, whereas $Z_{3}^{-1}$, $Z_{4}^{0}$, and $Z_{3}^{-3}$ were not significantly changed. In the TICL group, HOAs, $Z_{3}^{1}$ and $Z_{3}^{-3}$ were significantly increased compared to before surgery, whereas $Z_{3}^{-1}$, $Z_{4}^{0}$, and $Z_{3}^{3}$ were not significantly changed.

**Table 3:** Preoperative and postoperative aberration (µm) of eyes in the TICL and OCCI Groups^a^

Parameter	**TICL(n = 16)**^**c**^	**OCCI(n = 12)**^**c**^	*P*_*b*_
**HOAs**			
Preoperative	0.353 ± 0.075	0.399 ± 0.066	0.101
Postoperative (6 month)	0.387 ± 0.092*	0.527 ± 0.139*	0.003
$$Z_{3}^{1}$$			
Preoperative	0.037 ± 0.099	−0.006±0.178	0.423
Postoperative (6 month)	0.073 ± 0.089*	0.027 ± 0.181*	0.432
$$Z_{3}^{-1}$$			
Preoperative	−0.010±0.149	0.028 ± 0.199	0.563
Postoperative (6 month)	−0.013±0.158	0.029 ± 0.177	0.523
$$Z_{4}^{0}$$			
Preoperative	0.198 ± 0.112	0.208 ± 0.074	0.780
Postoperative (6 month)	0.237 ± 0.045	0.167 ± 0.121	0.077
$$Z_{3}^{3}$$			
Preoperative	0.001 ± 0.097	−0.001±0.079	0.828
Postoperative (6 month)	0.007 ± 0.077	0.112 ± 0.133*	0.026
$$Z_{3}^{-3}$$			
Preoperative	−0.035±0.097	−0.066±0.108	0.434
Postoperative (6 month)	0.030 ± 0.072*	0.019 ± 0.167	0.840

HOAs = higher order aberrations;TICL = toric Implantable Collamer Lens (STAAR Surgical); OCCI = opposite clear corneal incision

^a^Values are presented as mean ± standard deviation

* indicates significant difference between preoperative and postoperative values (＜0.05)

b means comparison between the TICL and OCCI groups

c HOAs were analyzed only in eyes with acceptable Pentacam imaging quality at the 6-month visit. Eyes with poor image centration, irregular tear film, or incomplete data within the 6-mm central corneal zone were excluded (TICL n=16,OCCI n=12)

## Discussion

OCCI has been widely used and recognized as an effective method to treat astigmatism in cataract surgery. Lever and Dahan reported that OCCI corrected an average astigmatism of 2.06D in cataract surgery [9]. To our knowledge, the present study is the first to directly compare ICL combined with bilateral penetrating OCCI versus TICL implantation specifically in eyes with moderate to severe astigmatism (1.5–3.0 D). The technique involves making a main penetrating incision at the meridian of the steep axis, followed by a full-thickness penetrating incision at the opposite meridian. The size of the double incisions is determined according to the magnitude of astigmatism. The shortcomings of OCCI are the risk of astigmatism regression as the incisions healed, and the increased risk of leakage or infection associated with double incisions [14]. However, none of the patients in our study developed intraocular infection after surgery, and the patients are still being followed up annually to observe its stability.

In this study, we evaluated the effectiveness of OCCI combined with ICL by assessing postoperative visual acuity, astigmatism, and higher-order aberrations. Figure 1 (A, B, C, D, E, F) shows that the OCCI group performed well in terms of visual acuity, accuracy of SEQ, predictability and stability, but was slightly inferior to TICL. Figure 1Gshows that the proportion of postoperative residual astigmatism within 0.5D or 1.0D in TICL was higher than that in OCCI (≤0.5D: 55% vs 45%; ≤1.0D: 95% vs 90%). It should be emphasized that the R^2^ value derived from the linear regression of TIA versus SIA (Fig. 1H) reflects only the strength of the linear association between the targeted and the surgically achieved astigmatic vectors, not the overall accuracy of the refractive correction. In the OCCI group, the paired penetrating incisions were performed according to an empirical nomogram. When under-correction occurs in a relatively systematic fashion (as reflected by the mean CI of 0.90), the data points may align along a consistent linear trend, yielding a numerically higher R^2^ (0.5615). Conversely, in the TICL group, the SIA values clustered tightly around the ideal target (mean CI close to 1.0). This near-complete correction with minimal residual variance paradoxically reduces the linear correlation coefficient, resulting in a somewhat lower R^2^ (0.4494). Importantly, with only 20 eyes per group, regression analyses are highly susceptible to the influence of individual data points, and a single outlier can substantially alter the R^2^. Therefore, R^2^ alone is an unreliable metric of refractive performance in this small-sample context. More clinically meaningful endpoints—including the proportion of eyes with residual cylindrical error ≤ 0.5 D (55% vs. 45%) or ≤1.0 D (95% vs. 90%), the mean CI (0.95 vs. 0.90), and UDVA outcomes—all consistently favored the TICL group. This is also consistent with the R^2^ of 0.8694 reported for TICL by Liu et al. in a larger cohort, which likely reflects more stable regression estimates [10]. We therefore base our comparative conclusions on these clinically relevant measures rather than on the R^2^ coefficient alone. There were no significant differences in TIA, SIA, DV, CI and AE between the two groups at postoperative 6 months (Table 2), but the proportion of OCCI within ±15° was lower than that of TICL (85% vs 100%) (Fig. 1I). It should be noted that the SIA in the TICL group (1.52 ± 0.51 D) closely approximated the TIA (1.60 ± 0.21 D). This pattern reflects the dominant contribution of the toric ICL’s refractive correction to the total astigmatic change, rather than incision-induced astigmatism from the small superior wound. Indeed, this magnitude of SIA is consistent with previous reports on toric ICL implantation for moderate astigmatism, where the cylindrical correction delivered by the lens itself accounts for the majority of the surgically induced vector. For example, Wei et al. reported mean SIA values of 1.78 ± 0.70 D and 2.11 ± 1.09 D for the V4c and V4 TICL models, respectively [15]. Similarly, Liu et al. reported a mean SIA of 1.46 ± 0.53 D following TICL implantation for moderate to severe astigmatism [10], and Yang et al. documented postoperative SIA values of 1.68 (1.26, 1.96) D [11]. Although our routine superior incision may have introduced a small degree of with-the-rule flattening, its influence on the overall SIA in the TICL group was minor. Future prospective studies using a temporal incision may help further isolate the effect of the incision from the lens-based correction. In terms of higher order aberrations, our results indicate that OCCI introduced more HOAs and $Z_{3}^{3}$ than TICL (Table 3). However, Zhou et al. reported that OCCI combined with ICL introduced more HOAs and $Z_{3}^{-3}$ than a single small incision combined with ICL [13]. Their study can be used as a reference because there is no significant difference in HOAs, coma, trefoil and spherical aberration caused by ICL implantation and TICL implantation with a small incision [15]. Although $Z_{3}^{3}$ and $Z_{3}^{-3}$ represent different orientations, they all belong to the trefoil family. When comparing the preoperative and postoperative results of the same group, OCCI led to a significant increase in HOAs, $Z_{3}^{1}$, and $Z_{3}^{3}$, while Zhou et al. reported that OCCI only led to a significant increase in HOAs and $Z_{3}^{-3}$ [13]. The discrepancy may be attributed to factors such as the inconsistency in the size of the double incisions determined by the degree of astigmatism, errors in incision symmetry, and irregularities in the wound surface. The HOAs, $Z_{3}^{1}$, and $Z_{3}^{-3}$ were significantly increased in the TICL group at 6 months after surgery. The increase of HOAs and $Z_{3}^{-3}$ is consistent with the research of Wei et al., whereas their research does not show a significant increase in coma [15]. This might be due to the factor that this study recorded the aberrations within 6 mm in diameter, while Wei et al. recorded within 5 mm in diameter. [15]. It has also been reported that $Z_{3}^{-3}$ and spherical aberration were significantly increased at 3 months after ICL surgery, while HOAs and coma showed no significant change [16]. Currently, there are few reports on high order aberrations caused by ICL surgery, and the results of these reports on high order aberrations are inconsistent. The discrepancies in specific trefoil components observed across studies highlight the inherent variability of the OCCI technique and underscore the need for standardized surgical protocols and consistent HOA measurement methodologies in future investigations. A larger sample size is needed to obtain definitive results.

Several limitations of this study should be acknowledged. First, this was a retrospective, non-randomized study with a relatively small sample size (20 eyes per group). The allocation of patients to the TICL or OCCI group was based on patient preference rather than random assignment, which may introduce selection bias. Although baseline characteristics were comparable between the two groups (Table 1), the possibility of unmeasured confounding factors cannot be entirely excluded. Second, the follow-up period of 6 months is relatively short to assess the long-term stability of astigmatism correction, particularly for the OCCI group where regression may occur as the incisions heal. Third, the sample size calculation was not performed a priori, and the study may be underpowered to detect small differences between groups.

Despite these limitations, this study provides valuable real‑world evidence on the comparative effectiveness of ICL combined with OCCI in the context of shared decision‑making. Nevertheless, patient self‑selection introduces inherent selection bias, and a longer follow‑up (≥1 year) is warranted to fully evaluate long‑term stability, particularly in the OCCI group where corneal wound healing may contribute to regression over time. Importantly, this study was not designed to demonstrate superiority; it offers preliminary evidence on the performance of this approach when TICL is not a preferred option. Prospective randomized controlled trials with larger sample sizes and longer follow‑up are needed to confirm our findings.

## Conclusions

Zhou et al. confirmed that ICL combined with OCCI has a significant effect on the treatment of mild to moderate astigmatism compared with no corneal steep axis release [13]. In this study, TICL was set as the control group and the subjects were patients with moderate to severe astigmatism to further verify the effect of OCCI in the treatment of astigmatism. In conclusion, OCCI is inferior to TICL in terms of visual acuity, refractive accuracy, and higher-order aberrations. This may be due to the fact that OCCI is based on an empirical formula, and its effectiveness in treating astigmatism is influenced by multiple factors such as the distance of the incision from the corneal center and the length of the tunnel. This is also why its results are less favorable compared to TICL. Despite the retrospective design and its limitations, our findings indicate that ICL combined with bilateral penetrating OCCI provides acceptable visual and refractive outcomes, supporting it as a viable alternative for patients with moderate to severe astigmatism who are not candidates for or decline TICL implantation.

## Data Availability

The datasets generated and/or analysed during the current study are not publicly available due to privacy restrictions or institutional policy, but are available from the corresponding author on reasonable request.

## Abbreviations

AE: Angle of error
AK: Astigmatism keratotomy
CCI: Clear corneal incision
CDVA: Corrected distance visual acuity
CI: Correction index
CRI: Corneal relaxing incisions
D: Diopter
DV: Difference vector
HOAs: Higher-order aberrations
ICL: Implantable Collamer Lens
IOL: Intraocular lens
logMAR: Logarithm of the minimum angle of resolution
OCCI: Opposite clear corneal incisions
SEQ: Spherical equivalent refraction
SIA: Surgically induced astigmatism
SPSS: Statistical Package for the Social Sciences
TIA: Target-induced astigmatism
TICL: Toric implantable Collamer Lens
UBM: Ultrasound biomicroscopy
UDVA: Uncorrected distance visual acuity
$$Z_{4}^{0}$$: Spherical aberration
$$Z_{3}^{1}$$: Horizontal coma
$$Z_{3}^{-1}$$: Vertical coma
$$Z_{3}^{3}$$: Horizontal trefoil
$$Z_{3}^{-3}$$: Oblique trefoil
